# Aqueous Humor Levels of Vascular Endothelial Growth Factor and Stromal Cell-Derived Factor-1α in Age-Related Macular Degeneration

**DOI:** 10.14744/bej.2021.49140

**Published:** 2021-12-17

**Authors:** Ali Keles, Yasemin Ozdamar Erol, Sema Nur Ayyildiz, Suleyman Korhan Karaman, Elmas Ogus

**Affiliations:** 1. Department of Ophthalmology, Bilecik Seyh Edebali University Faculty of Medicine, Bilecik, Turkey; 2. Department of Ophthalmology, University of Health Science, Ulucanlar Eye Training and Research Hospital, Ankara, Turkey; 3. Department of Medical Biochemistry, Ankara Training and Research Hospital, Ankara, Turkey

**Keywords:** Age-related macular degeneration, aqueous humor, stromal cell-derived factor-1α, vascular endothelial growth factor

## Abstract

**Objectives:**

This study investigated the contribution of vascular endothelial growth factor (VEGF) and stromal cell-derived factor-1α (SDF-1α) angiogenic mediators in eyes with age-related macular degeneration (AMD).

**Methods:**

Aqueous humor specimens taken during cataract surgery in 7 cases of intermediate stage (nonexudative) AMD and 7 cases of late stage (exudative) AMD were evaluated using chemiluminescent immunoassay testing in this prospective case-control study. Mediator levels were compared with the normal reference values of 7 patients without any disease other than cataract.

**Results:**

The groups were similar in terms of age and gender (p>0.05). The aqueous humor levels of VEGF in both the intermediate AMD (median: 224.3 pg/mL, range: 44.8–380.4 pg/mL) and late-stage AMD (median: 108.7 pg/mL, range: 61.9-223.5 pg/mL) patients were similar to those of the control group (median: 121.1 pg/mL, range: 24.9–156.6 pg/mL) (p=0.256). Moreover, there was no significant difference in the SDF-1α concentrations between the intermediate AMD (median: 160.9 pg/mL, range 130–166.3 pg/mL), late AMD (median: 161 pg/mL, range: 154.1.9–171.6 pg/mL), and control group values (median: 161 pg/mL, range: 155.2–219 pg/mL) (p=0.763).

**Conclusion:**

The aqueous humor levels of VEGF and SDF-1α were within the normal range in patients with intermediate and late-stage AMD.

## Introduction

Age-related macular degeneration (AMD) is the most common cause of blindness in Europe with 26% (1). The prevalence of AMD is 9–25% in the 65–75 age group (2). Early stages are classified into early AMD with medium-sized drusen (≥63–<125 μm drusen without pigmentary abnormalities) and intermediate AMD marked by large drusen (>125 microns and/or drusen associated with pigmentary abnormalities). Late AMD is associated with a reduction in central vision and has two forms: atrophic (dry) AMD characterized by geographic atrophy and exudative (wet) AMD characterized by choroidal neovascularization (CNV) (3).

Angiogenesis and inflammation induced by an unknown cause result in CNV formation in the exudative type AMD pathogenesis responsible for 90% of AMD related blindness (4). Newly-formed vessels originating from the choroid, with blood and fluid, break through the Bruch’s membrane into the subretinal pigment epithelial space and/or the subretinal space, and irregular elevations occur on the surface of the retina (5). Experimental and clinical evidences have shown that vascular endothelial growth factor (VEGF) is the key component in promoting neovascularization (6). Intravitreal anti-VEGF agents have dramatically improved visual results (7, 8). On the other hand, despite standardized anti-VEGF therapy, persistent fluid or recurrent exudate continues to form in the eyes of some patients (7, 9).

There are many reasons for the decreased response to anti-VEGF treatment: initiation of treatment at the chronic stage, misdiagnosis, low initial visual acuity, metabolic causes, genetic variation, and tachyphylaxis. There may be changes in absorption, distribution, or metabolism reducing the effective concentration of the drug. Cellular mechanisms include decreased number of drug receptors, decreased concentration, or declined binding to drug receptors (10). In addition, response to anti-VEGF may vary due to upregulation of cytokines/molecules other than VEGF (eg, Hepatocyte growth factor, Fibroblast growth factor, Platelet-derived growth factor, E-Selectin, and Intercellular Adhesion Molecule 1), activation of the complement system, or inflammation in CNV lesions (11, 12). This situation may explain the response of some CNV lesions to the complementary treatment of triamcinolone (13). In addition, chronic anti-VEGF treatment has been reported to be associated with the development of retinal atrophy in some patients with AMD (14).

The presence of persistent CNV despite anti-VEGF therapy indicates gaps in understanding basic biological mechanisms. For alternative therapeutic options for CNV, there is a need to identify mediators other than VEGF and to determine the extent to which these factors contribute to CNV development. Stromal cell-derived factor-1α (SDF-1α), otherwise known as C-X-C motif chemokine 12 (CXCL12) (15), which is mainly known as the chemotactic factor for hematopoietic progenitor cells, is expressed in response to tissue ischemia (16) and provides angiogenetic potency (17).SDF-1α does not show proliferation effect in the absence of exogenous VEGF (17).

Based on the idea that other factors besides VEGF may be influential, we evaluated the aqueous humor (AH) levels of VEGF and SDF-1α mediators in eyes with different stages of AMD and compared these results with healthy control group.

## Methods

This case-control designed study was carried out at a tertiary hospital in accordance with the ethical standards of the Declaration of Helsinki. The study protocol was approved by the local ethics committee.

The present study comprised 21 eyes of 21 adult patients (11 eyes of 11 male patients and 10 eyes of 10 female patients) undergoing cataract surgery. All patients underwent a complete ophthalmic examination and optical coherence tomography (OCT). The study group consisted of 14 eyes with AMD and the control group consisted of seven eyes with undergoing routine cataract surgery. Control subjects had no systemic or ocular pathology. Patients with diabetes mellitus, glaucoma, uveitis, history of vitreoretinal surgery, and intravitreal injection within the past 3 months were excluded from the study. The patients with AMD were divided into two subgroups: seven cases of intermediate (nonexudative) AMD and seven cases of late (exudative) AMD. The intermediate AMD group consisted of the cases with marked by large drusen (>125 microns and/or drusen associated with pigmentary abnormalities), and the late AMD group consisted of cases with CNV scarring (3). There was no activity detected by clinical examination, fluorescein angiography and OCT in eyes with late AMD within the past 3 months. In late AMD cases, CNV was in the form of fibrovascular scar tissue.

### Assessment of AH VEGF and SDF-1α Levels

Undiluted mean volume of 0.2 mL of AH samples was obtained by anterior chamber limbal paracentesis with a 30-G needle attached to an insulin syringe just before starting phacoemulsification procedure. Injection of viscoelastic agent was subsequently performed through anterior chamber to form volume and then operation was continued. After the collection, AH samples were immediately placed on ice and transferred to Training and Research Hospital Medical Biochemistry Laboratory. Therein, the samples were centrifuged at 10000 rpm for 10 min, then separated into two sterile eppendorf tubes and stored at –80°C until analyzed. AH samples were analyzed by enzyme-linked immunosorbent assay (ELISA) to determine the concentration of VEGF and SDF-1α. This evaluation was done in the both research groups and the control group.

AH samples were thawed at room temperature and vortexed. AH VEGF and SDF-1α levels were employed by ELISA method using kits for human VEGF (R and D Systems, Minneapolis, MN, USA) and human SDF-1α (R and D Systems, Minneapolis, MN, USA). The El × 50 automatic strip washer (BioTek Instruments, USA) was used in the study and optical density was measured at 450 nm with EL × 808 Brand ELISA reader (BioTek Instruments, USA). Each assay was performed according to the manufacturer’s instructions and used 100 μl AH sample for the VEGF ELISA and 100 μl for the SDF-1α ELISA.

### Statistical Analysis

Descriptive statistics were examined as mean ± standard deviation or median with range. The normal distribution of the variables was tested using visual and analytical methods. One-way analysis of variance test was used as the statistical method for normally distributed data. For data that showed nonparametric distribution, Kruskal–Wallis test was applied to determine whether there was any significant difference between the three groups. P<0.05 were accepted as significant. Analysis of the data was performed in the SPSS 22.0 (Chicago, IL, USA) package program.

## Results

Patient demographics and concentration of VEGF and SDF-1α in AH fluids of patients with AMD and control subjects are shown in Table 1 . No significant difference was observed among the groups with respect to age and gender (p=0.085 and p=0.417, respectively; (Table 1)). There was no significant difference in VEGF levels between intermediate AMD (median 224.3 pg/ml, range 44.8–380.4), late AMD (median 108.7 pg/ml, range 61.9–223.5), and control groups (median 121.1 pg/ml, range 24.9–156.6), (p=0.256, (Table 1)). Furthermore, the difference in the the SDF-1α levels between intermediate AMD (median 160.9 pg/ml, range 130–166.3), late AMD (median 161 pg/ml, range 154.1.9–171.6), and control groups (median 161 pg/ml, range 155.2–219) was not significant (p=0.763, (Table 1)).

**Table 1.** Demographic and clinical characteristics of patients according to age-related macular degeneration and control groups

	**Intermediate AMD (n=7)**	**Late AMD (n=7)**	**Control (n=7)**	**p-value**
Age, years, Mean±SD	72.1±4.6	73.0±12.2	63.7±5.1	0.085^*^
Sex, (Male/Female)	4/3	5/2	2/5	0.417^†^
VEGF, pg/ml, Median (Min-Max)	224.3	108.7	121.1	
	(44.8–380.4)	(61.9–223.5)	(24.9–156.6)	0.256^‡^
SDF-1α, pg/ml, Median (Min-Max)	160.9	161	161	
	(130–166.3)	(154.1–171.6)	(155.2–219)	0.763^‡^

N: Number; SD: Standard deviation; AMD: Age-related macular degeneration. ^*^One-Way ANOVA test. ^†^Fisher’s exact probability test. ^‡^Kruskal-Wallis test.

## Discussion

Angiogenesis, a multi-step process, is a key mechanism for the development of CNV in AMD. CNV is induced by impaired dynamic balance between positive and negative regulators in angiogenesis (5). The main aim of the study was to understand the role of VEGF and SDF-1α in CNV related late stage (exudative) AMD pathophysiology. The reason we looked at these mediators in the non-exudative type was to investigate whether the possible significant change occurred in the early stages AMD. However, the results showed no difference VEGF and SDF-1α levels in AH of patients with intermediate stage (nonexudative) and late stage (exudative) AMD versus controls.

VEGF supports abnormal vascular proliferation in AMD (18). Except for the obvious difference in the study of Tong et al. (19), various studies showed that VEGF concentrations in AH of CNV secondary to AMD slightly increased versus controls (20-22). However, several studies found no significant difference in AH levels (23-26). Furthermore, Huber and Wachtlin (27), did not notice a difference in VEGF vitreous fluid levels of patients with CNV and control group. They asserted that significantly higher soluble VEGF receptor 1 levels in the exudative AMD group compared to controls may be due to increased permeability of blood retinal barriers (27). Mimura et al. (28) reported that AH levels of VEGF, soluble VEGF receptor 1, soluable VEGF receptor 2 and inflammatory factors were significantly higher in the AMD/CNV group versus control. Abnormal vessels with porous walls give rise to subretinal hemorrhage or fluid leakage in CNV eyes (28). Muether et al (25). mentioned that the AH of VEGF level was interestingly elevated in intermediate dry AMD but not in exudative AMD. They commented that elevated VEGF levels in intermediate AMD may be due to complement activation and inflammation through drusen, and VEGF contributes to CNV formation as the primary angiogenic signal, but VEGF levels do not directly reflect CNV activity (25). In our study, we detected high VEGF levels in eyes with intermediate (nonexudative) AMD, but the difference was no statistically significant. We suppose that VEGF may play a role in the pathogenesis of non-exudative AMD. In addition, we found no significant difference about VEGF levels between late stage (exudative) AMD and controls. The fact that late AMD cases were clinically inactive may contribute to this result.

Studies on diabetic retinopathy characterized by neovascularization due to retinal ischemia have shown that SDF-1 plays an important role (29-31). Even Chen et al. (30) found a significant positive correlation between SDF-1 and VEGF in the vitreous of proliferative diabetic retinopathy patients. However, Keles et al. (31) observed no relationship between SDF-1 and VEGF in the vitreous of active proliferative diabetic retinopathy patients. Further, the contribution of SDF-1 has been shown in the pathogenesis of retinopathy of prematurity characterized by angiogenesis (32). Furthermore, upregulation of SDF-1 expression was observed in a rat model of retinal ischemia-reperfusion injury, and microvasculature endothelial cells were specified to be potential role to SDF-1 production in ischemic retina (33). Besides, it was suggested in a study that SDF-1 has a crucial role in angiogenic changes during retinal vein occlusion, due to the higher intravitreal SDF-1 levels in active retinal vein occlusion compared to quiescent retinal vein occlusion and control groups (34).

Sakamoto et al. (35) showed that SDF-1α (CXCL12) level in AH was higher in the neovascular AMD group compared with controls, and concentration of SDF-1α decreased significantly after two anti-VEGF injections in patients with AMD. They hypothesized that anti-VEGF drugs inhibit VEGF signalling and VEGF- induced chemotaxis, thus reducing the inflammatory reaction (35). Zhou et al. (36) investigated several angiogenic and inflammatory cytokines from neovascular AMD patients, polypoidal choroidal vasculopathy patients and cataract patients, and stated that VEGF-A and SDF1-α were significantly higher in eyes with neovascular AMD andpolypoidal choroidal vasculopathy patients than controls. VEGF and SDF-1α may considerably play a role in clinically active CNV. We couldn’t elucidate this situation because we couldn’t get samples from patients with active CNV because of surgery-induced inflammation.

Ecker et al. (37) suggested that AH levels of some cytokines reflect their vitreous levels, while majority shows no correlation. Previous studies have reported corresponding AH and vitreous levels of VEGF in diabetic retinopathy and central retinal vein occlusion (38, 39). However, such data of VEGF is not available for AMD patients. Moreover, there is no broadcast notification for SDF-1α. AH level of VEGF and SDF-1α may directly not reflect the vitreous level in CNV, which is a small lesion compared to ocular glob. If vitreous fluid can be taken from the area close to the CNV lesion; angiogenic cytokines, including VEGF, can be measured at a higher concentration (24). Indeed, VEGF was detected in surgically extracted AMD-related CNV (40).

A small number of the patients is mainly missing aspect of research. Therefore, additional studies involving increased number of patients may help in determining a reliable precise difference in AH mediator levels. Consequently, there was no difference in VEGF and SDF-1α parameters between the patients diagnosed with intermediate AMD, late AMD (possessing clinically non-active CNV) and the control group in our study. It can be interpreted that clinical inactivity is confirmed by mediator level inactivity. To better understanding of AMD pathogenesis, various mediators should be investigated in different stages of AMD with larger sample sizes.

### Disclosures

**Ethics Committee Approval:** Ankara Numune Training arqnd Research Hospital Ethics Com- mittee, protocol number: E-17-1406, Date: 25/05/2017.

**Peer-review:** Externally peer-reviewed.

**Conflict of Interest:** None declared.

**Authorship Contributions:** Involved in design and conduct of the study (AK, YOE, SNA, SKK, EO); preparation and review of the study (AK, YOE); data collection (AK, YOE, SNA, SKK, EO); and statistical analysis (AK, YOE).
